# Elevated OPN, IP-10, and Neutrophilia in Loop-Mediated Isothermal Amplification Confirmed Tuberculosis Patients

**DOI:** 10.1155/2014/513263

**Published:** 2014-10-15

**Authors:** Beata Shiratori, Susan Leano, Chie Nakajima, Haorile Chagan-Yasutan, Toshiro Niki, Yugo Ashino, Yasuhiko Suzuki, Elisabeth Telan, Toshio Hattori

**Affiliations:** ^1^Division of Disaster-Related Infectious Diseases, International Research Institute of Disaster Science, Tohoku University, 1-1 Seiryo-machi, Aoba-ku, Sendai, Miyagi 980-8574, Japan; ^2^Division of Emerging Infectious Diseases, Graduate School of Medicine, Tohoku University, 1-1 Seiryo-machi, Aoba-ku, Sendai, Miyagi 980-8574, Japan; ^3^Japan International Corporation of Welfare Services, 2-3-20 Toranomon YHK Building 4F, Toranomon, Minato-ku, Tokyo 105-0001, Japan; ^4^STD AIDS Cooperative Central Laboratory, San Lazaro Hospital, Quiricada Street, 1003 Manila, Philippines; ^5^Division of Global Epidemiology, Research Center for Zoonosis Control, Hokkaido University, North 20, West 10 Kita-ku, Sapporo 001-0020, Japan; ^6^Research Division, GalPharma Company, Ltd., NEXT-Kagawa 204, 2217-44 Hayashi-cho, Takamatsu-shi, Kagawa 760-0301, Japan; ^7^Division of Disaster-Related Infectious Diseases, International Research Institute of Disaster Science, Tohoku University, 2-1 Seiryo-machi, Aoba-ku, Sendai 980-8574, Japan

## Abstract

Tuberculosis (TB) is the second most common cause of death from infectious diseases and results in high socioeconomic losses to many countries. Proper diagnosis is the first step in TB eradication. To develop a rapid, simple, and accurate diagnostic TB test and to characterize the prevalence of *Mycobacterium tuberculosis* (MTB) genotypes and immune profiles of TB patients, a total of 37 TB patients and 30 healthy control (HC) from Metro Manila were enrolled. Loop-mediated isothermal amplification (LAMP) reliably detected MTB infection. Manila genotype was identified by spoligotyping method in all TB patients. Osteopontin (OPN), interferon-*γ*-induced protein 10 kDa (IP-10), and neutrophil counts were found to reflect the acute stage of MTB infection. The sensitivity and specificity were 94.6% and 93.3%, respectively, for both OPN and IP-10, and they were 83.8% and 78.6%, respectively, for neutrophils. The combination of OPN, IP-10, neutrophil count, IL-6, IL-8, TNF-*α*, MCP-1, platelets, galectin-9, and leukocyte count correctly identifies all the HC and 96.3% of TB patients. LAMP method may serve as a rapid, supportive method in addition to time-consuming culture methods. OPN, IP-10, and neutrophil counts are useful in detecting MTB infection and may have utility in monitoring the course of the disease.

## 1. **Introduction**

*Mycobacterium tuberculosis* (MTB) is the causative agent of tuberculosis (TB), the sixth leading cause of death and illness in the Philippines. Despite global trends towards decreasing incidence, prevalence, and mortality associated with MTB infection, approximately 230,000 cases were found in the Philippines alone in 2012 [1]. The elderly, urban poor, smokers, and individuals with compromised immune systems, such as those living with HIV, malnutrition, and diabetes, belong to the high-risk group [2]. Beyond the health burdens associated with MTB, serious socioeconomic losses are another consequence of this disease.

Developing a rapid, simple, and accurate test for TB diagnosis is a main focus of many investigators. In low-resource countries, TB is most often diagnosed based on clinical symptoms, radiographic features, and microscopic observation of acid fast bacillus (AFB). Bacterial culture methods require long culturing time to obtain acceptable sensitivity, which delays early initiation of anti-TB treatment. In recent years, nucleic acid amplification (NAA) tests have shown potential as the optimal TB diagnostic approach for MTB diagnosis. Loop-mediated isothermal amplification (LAMP) is an NAA method that enables the detection of trace amounts of bacterial DNA under isothermal conditions within one hour [3].

Osteopontin (OPN) is a multifunctional phosphorylated glycoprotein that is synthesized by a variety of immune and nonimmune cells [4, 5]. Increased levels of OPN have been observed during MTB infection and other infectious pathogens [4, 6, 7], polarizing the immune response towards a T_h_1 response through the enhancement of IL-12 and IFN-*γ* secretion [4, 8].

Galectin-9 (gal-9) is a *β*-galactoside-binding matricellular protein that induces cell activation, chemoattraction, and cell death. Gal-9, binding to its receptor, T-cell immunoglobulin  and mucin domain-containing molecule-3 (Tim-3), stimulates bactericidal activity in mouse TB models [9, 10]. Gal-9 and Tim-3 expression in CD4+ and CD8+ T-cells may be elevated during TB infection in humans, compared to healthy individuals [11]; however, their concentrations in plasma have not been reported until now.

Interferon-*γ* induced protein 10 kDa (IP-10) is one of the most well studied biomarkers in TB infection and is a promising alternative marker for replacing current interferon-*γ* releasing assay-based methods [12]. IP-10 is involved in multiple biological functions, inducing chemotaxis, apoptosis, inhibition of cell growth, and recruiting activated T-cells, macrophages, and NK cells to sides of infection [12].

Relatively little attention has been paid to the role of neutrophils during MTB infection, compared to macrophages and other host immune response components. Neutrophils elicit strong phagocytic activity [13] and neutrophil-driven, interferon (IFN)-inducible transcript signatures in whole human blood, which were recently shown to be associated with disease severity, suggesting a direct contribution of neutrophils to pathogenesis [14]. Other studies have demonstrated that neutrophils contribute to early defense responses against MTB [15, 16], but in later stages of the disease, an opposite tendency is observed [17, 18].

In the present study, we evaluated the reliability of LAMP for detecting MTB infection and used spoligotyping to identify the most prevalent MTB genotype in Metro Manila. We also analyzed a broad spectrum of biomarkers, which reflect both cellular and humoral immune response to MTB infection. Our results confirmed the utility of the LAMP and spoligotyping methods for TB diagnostics and genotyping and showed that OPN, IP-10, and neutrophil counts reflect the acute stage of disease and are promising biomarkers to monitor the course of the disease.

## 2. Materials and Methods

### 2.1. Participants

The study population consisted of 37 HIV-negative patients randomly selected from the out-patient department of San Lazaro Hospital (SLH, Manila, Philippines) who had positive AFB staining, clinical symptoms, and chest radiographs characteristic of pulmonary TB and had no prior history of TB treatment. The controls were healthy volunteers lacking signs of TB; however, their histories of latent tuberculosis infection (LTBI) or other pulmonary diseases are unknown. The study was approved by the ethics committee of SLH and the Tohoku University Hospital, and written informed consent was obtained from each participant.

### 2.2. Sample Collection

Blood was collected in 5 mL EDTA tubes and plasma was separated by centrifugation and stored at −80°C until analyzed. Sputa were decontaminated by conventional procedures using NALC-NaOH. After centrifugation, supernatants were discarded and DNA was released from cell pellets by heating at 95°C for 5 min, which was repeated three times.

### 2.3. Clinical Data and Biomarker Measurement

Complete blood counts (CBC), including differential counts of white blood cells and plasma levels of IgG and IgA, were performed for samples from each individual. Plasma levels of 29 cytokines and chemokines were measured using the Milliplex MAP (Millipore, Germany). The Bio-Plex Manager Software (version 6.0) was used for bead acquisition and analysis. OPN protein levels were measured using the Human Osteopontin Quantikine ELISA Kit (R&D Systems, USA) and gal-9 levels by an ELISA kit (GalPharma, Japan). Antitubercular glycolipid (TBGL) IgG and IgA antibody titers were obtained using the Determiner TBGL Antibody ELISA Kit (Kyowa Medex, Japan).

### 2.4. LAMP Method

LAMP reactions were performed in reaction mixture (25 *μ*L) containing 6 primers [3], 1.4 mM deoxynucleoside triphosphates, 0.8 M betaine, 20 mM Tris/HCl (pH**8.8), 10 mM KCl, 10 mM (NH_4_)_2_SO_4_, 8 mM MgSO_4_, 8 U* Bst* DNA polymerase (New England Biolabs), and 5 *μ*L of bacterial DNA. The mixture was incubated at 64°C for 60 min in a Loopamp real-time turbidimeter (LA-200, Teramecs). Reactions were considered positive when turbidity was greater than 0.1 (650 nm) within 60 min.

### 2.5. Spoligotyping Method

Spoligotypes of clinical MTB isolates were determined as described previously [19]. Briefly, the DR region was amplified with a primer pair, and the PCR products were hybridized to a set of 43 spacer-specific oligonucleotide probes, which were covalently bound to membranes. The spoligo-international type was determined by comparing spoligotypes against the international spoligotyping database [20].

### 2.6. Statistical Analysis

The Mann-Whitney *U*-test was used to compare the differences between the two groups, and the Spearman's rank correlation was used to analyze relationships between biomarkers and other parameters. Statistical analyses were performed using the GraphPad Prism program (version 6, GraphPad Software, USA) and the SPSS program (version 22, SPSS Inc., USA). Discriminant function analyses and Receiver operator characteristic (ROC) curve analyses were used to evaluate the predictive capacities of biomarkers. Area under the curve (AUC) values were calculated, and cut-off values were estimated based on the best proportion between sensitivity, specificity, and likelihood ratio.* P* values < 0.05 were considered statistically significant.

## 3. Results

### 3.1. Characteristics of Study Participants

A total of 37 TB patients and 30 healthy controls (HC) were enrolled in this study. Basic anthropometric and clinical characteristics of study participants are shown in Table 1. Chest lung X-rays showed bilateral pathology in 68.8% of cases, and only one lung was affected in 31.2% of cases. Lung cavities and effusion were found in 47% and 6.3% of TB patients, respectively. Laboratory findings revealed lower red blood counts, hemoglobin levels, hematocrits, and relative lymphocyte counts in the TB group compared to the HC group. Absolute numbers of white blood cells (WBC) and relative counts of neutrophils, monocytes, platelets, and plasma IgG and IgA were higher in TB patients (Table 1). The absolute and relative values of each type of WBC were strongly correlated (data not shown).

### 3.2. LAMP and Genotyping

We confirmed MTB infection in all 37 patients' samples by the LAMP method. All DNA samples were subjected to spoligotyping to identify the most prevalent genotype. We found that all patients were infected with a Manila type MTB.

### 3.3. Comparison of Plasma Biomarker Concentrations between TB and HC Individuals

Using a multiplex immunoassay, we assessed the concentrations of 29 soluble plasma biomarkers in HC, as well as TB patients before antituberculous therapy was initiated. Biomarker levels and their comparisons between groups are shown in Table 2. Significantly elevated concentrations of IL-1*α*, TNF-*α*, IL-6, eotaxin, IL-8, IP-10, and MCP-1 were observed with the TB group. IL-1*β*, IL-2, IL-3, IL-15, IL-17A, IL-4, IL-5, IL-13, MIP-1*α*, TNF-*β*, and IL-10 levels were not considered for statistical analyses because their median was below minimum detectable levels in both groups. We measured also the plasma levels of two matricellular proteins, OPN and gal-9, and found statistically higher levels of both proteins in TB patients compared to HC individuals. Higher titers of TBGL IgG and IgA were found in TB patients. In HC, antitubercular antibodies were correlated with age, anti-TBGL IgG (*r* = 0.66, *P* < 0.00001), and IgA and (*r* = 0.327, *P* < 0.05); however, this relationship was not observed in the TB group (data not shown). Anti-TBGL IgG correlated with anti-TBGL IgA (*r* = 0.333, *P* < 0.05) in the TB group but not in the HC group (data not shown). Anti-TBGL antibody elevation was not related to a general elevation of antibodies in the plasma (data not shown).

### 3.4. Correlation of OPN, IP-10, Neutrophil Count, and Gal-9 in HC and TB Groups

We performed correlation analyses of the three most predictive TB markers with other measured parameters. IP-10 expression levels positively correlated with loss of appetite, IL-1*α*, IL-6, IL-8, OPN, and gal-9 expression and negatively correlated with anti-TBGL IgG antibody titer (Table 3). In addition, OPN and IL-8 expression levels were correlated (Table 3). TB patients' neutrophilia was associated with increased WBC counts, IFN-*γ*, and decreased hemoglobin levels, hematocrits, plasma IgG titers, and lymphocytopenia (Table 3).   Finally, we studied the relationship of gal-9 to biomarkers and other laboratory parameters. High gal-9 levels correlated with higher TNF-*α*, IL-6, IL-5, IL-3, EGF, IL-8, and IP-10 expression and lower levels of GM-CSF (Table 3).

### 3.5. Discrimination Potential of Biomarkers

Biomarkers that differentiated HC and TB were analyzed by ROC analysis and cut-off values were determined based on the best proportion between sensitivity, specificity, and likelihood ratio (Table 4). The highest discriminatory property had IP-10 and OPN, followed by neutrophils, platelets, TNF-*α*, MCP-1, leukocyte counts, gal-9, and IL-8. Next, we performed analysis, which is a multivariate discrimination method to characterize the most discriminatory variables among groups. OPN was identified as having the highest discriminatory capacity, followed by IP-10, neutrophils, IL-6, IL-8, TNF-*α*, MCP-1, platelets, gal-9, and WBC. Using a combination of these markers, all healthy individuals and 96.3% of TB patients were correctly classified.

## 4. Discussion

Despite its low sensitivity, AFB staining is often used in attempt to differentiate mycobacterial infection from other pulmonary bacterial infections. Bacterial culture methods, the QuantiFERON TB test, and PCR have improved the sensitivity and sensitivity of TB detection but are time-consuming and require trained laboratory workers. Recently, we developed a LAMP method for detecting MTB with excellent accuracy in one hour [3]. Here, we confirmed MTB infection in all of our samples by LAMP; bacterial cultures were not performed because previous studies showed that MTB-LAMP sensitivity in culture positive samples reached 100% and that specificity in culture negative samples was 94.2% [3]. Moreover, the research group from Thailand found that, in the clinical unknown samples test, the sensitivity of LAMP method was 98.92% and the specificity was 100% compared to those of the standard culture assay [21]. Samples were also studied by spoligotyping to determine the most prevalent circulating strains in Metro Manila. We found that all of our TB patients were infected with a Manila type of MTB and this finding of a uniformed genotype of MTB was rather surprising. The arrival of Chinese, Japanese, and Spanish groups may have influenced the acquisition of various MTB genotypes in the region; however, little information is available regarding prevalent MTB genotypes circulating in the Philippines.

The role of matricellular proteins supporting TB infection is not well studied. We found high plasma levels of two matricellular proteins, OPN, and, for the first time, gal-9 in treatment-naïve TB patients. Matricellular proteins are secreted into the extracellular matrix environment but do not play a primary structural role in this location and regulate an unusually diverse array of cellular functions, including cell adhesion, shape, migration, differentiation, proliferation, and inflammatory responses [22]. It has been proposed that matricellular proteins enter inflamed tissue and become immobilized at that site to generate signals for phagocytosis and chemotaxis of inflammatory cells [23]. OPN is highly expressed in tuberculous granuloma and supports granuloma formation via its functions as a chemoattractant cytokine [24]. OPN and gal-9 may be produced by activated circulating immune cells, but a more plausible explanation is that they are released into the circulation from tissue sites. Our unpublished data showed elevated pleural fluid/plasma ratios of gal-9 and OPN in a TB patient, and Inomata et al. showed that OPN levels increase proportionally with the extent of lung lesions [25]. We evaluate differences between patients with and without granuloma formation, factoring in the extent of lesions and other clinical parameters; however, we did not observe any statistical correlations, probably because radiological findings with most patients were not suggestive of granulomas. We found that the OPN and gal-9 correlated with IL-8 and IP-10 and we hypothesize that OPN and gal-9 activate the expression of chemokines and cytokines like IP-10, TNF-*α*, IL-6, and IL-8 in macrophages, helping to recruit immune cells to the site of MTB infection. This speculation is supported by observations from Bai et al., who found that matricellular protein CCN1 enhances the expression of TNF-*α*, IL-1*β*, IL-6, IFN-*γ*, MIP-1*α*, and IP-10 in murine macrophages through its receptors *α*_M_ *β*_2_ and syndecan-4 [22]. IP-10 is produced at sites of inflammation and its blood levels reportedly correlate with the extent of inflammatory process [26, 27]. In contrast with low circulating levels of IFN-*γ*, the high levels of IP-10 in the blood produced by antigen-stimulated cells make this chemokine a promising candidate biomarker for MTB infection, even in HIV-infected individuals [28, 29]. We found that patients with high plasma IP-10 reported loss of appetite, but such a tendency was not observed with other biomarkers. Juffermans et al. had similar observations, where IP-10 but not IL-8, MIP-1b, or MCP-1 was associated with loss of appetite and fever [30].

Neutrophils, one of the most predictive markers of active TB infection, were found to have the largest influence on WBC elevation in TB patients. Neutrophils are generally thought to have strong activity against infectious agents. Higher neutrophil counts were observed in TB patients and were associated with poorer prognosis [31]. Neutrophils are attracted by various cytokines and chemokines, including IL-8, and quickly accumulate at sites of mycobacterial infection [32–34]. We also observed IL-8 elevation in TB patients, which may be due to hyperproduction by stimulated macrophages, by epithelial cells in lungs, or even by neutrophils themselves, although no correlation with neutrophils was observed. Previous study showed that low lymphocyte count is suggestive of extension of the disease by new tubercle formation, but switch to lymphocytosis shows tendency to healing [35]. Therefore, careful evaluation of hematological changes may have diagnostic value.

It is generally accepted that cell-mediated immunity has a critical role in the protection against MTB. However, MTB is a facultative intracellular pathogen, which has both intracellular and extracellular phases in their infectious cycle. Recent studies showed that immunization using monoclonal and polyclonal antibodies and mucosal vaccination trials demonstrate convincingly the essential interdependence and synergy between cell-mediated immunity and humoral immunity [36]. WHO warned against the utility of current serological tests in the immunodiagnosis of TB and strongly recommended that they must not be used for the diagnosis of pulmonary and extrapulmonary TB, but we believe that detection of anti-TBGL antibody titers may serve as a warrant signal of MTB infection.

In our study, TB patients represented a small group of individuals who underwent the examination due to suspected TB disease. In all of the patients, only Manila genotype was identified; therefore, it would be of interest to study the immunological profile of TB patients infected by other MTB genotypes. Future, larger sample size studies should prove the application of  LAMP method and proposed biomarkers in TB research.

In conclusion, LAMP is a simple and low-cost method for the direct detection of MTB in patients' sputa. Moreover, the LAMP method together with spoligotyping method serves as sensitive, accurate tools for TB diagnosis and for monitoring prevalent lineages of TB in certain regions. Increased IP-10, OPN, and neutrophils levels best reflect the acute stage of TB infection, and measuring their fluctuations may provide a reasonable basis for determining TB severity and prognosis.

## Figures and Tables

**Table 1:** Characteristics of HC and TB individuals.

	HC (*n* = 30)	TB (*n* = 37)	*P* value
Antropometric data			
Age year; median (range)	28.5 (22–59)	40 (18–60)	ns
Gender: male; *n* (%)	9 (30)	23 (62.2)	∗∗
BMI	na	20.42 (14–44.5)	
Employed (%)	100	48.4	
Had contact with TB (%)	0	48.6	
Clinical symptoms			
Cough (%)	na	94.4	
Fever (%)	na	77.8	
Sweat (%)	na	66.7	
Loss of appetite (%)	na	25	
Radiographic features			
Site of pathological finding			
One lung (%)	na	31.2	
Both lungs (%)	na	68.8	
Cavity (%)	na	46.9	
Effusion (%)	na	6.3	
Laboratory findings: median (range)			
RBC (10^6^/*μ*L)	4.8 (4.3–6.5)	4.6 (3–6.2)	∗
Hemoglobin (g/dL)	13.8 (12.2–17.2)	12.5 (8.3–16)	∗∗∗∗
Hematocrit (%)	42.2 (39.1–51.8)	37.8 (25.6–49.6)	∗∗∗∗
WBC (10^3^/*μ*L)	6.8 (4.4–11.8)	9.1 (4.4–19.6)	∗∗∗∗
Neutrophil (%)	52.5 (37–67)	70 (49–85)	∗∗∗∗
Lymphocyte (%)	38.5 (24–68)	19 (6–33)	∗∗∗∗
Monocyte (%)	5 (2–11)	8 (3–13)	∗∗∗∗
Eosinophil (%)	3.5 (1–8)	3 (1–10)	ns
Platelet (10^3^/*μ*L)	305.5 (178–560)	467 (143–784)	∗∗∗∗
Plasma IgG (mg/dL)	1134 (659–3266)	1618 (934–2456)	∗∗
Plasma IgA (mg/dL)	329 (115–973)	512 (162–1126)	∗∗

^*^ *P* < 0.05, ^**^ *P* < 0.01, ^***^ *P* < 0.001, ^****^ *P* < 0.0001.

TB group versus HC group by Mann-Whitney *U*-test.

ns: not significant, na: not applicable.

**Table 2:** Comparison of cellular and humoral immunity biomarkers between HC and TB individuals.

Biomarker	HC	TB	*P*
(pg/mL)	Median (range)	
General activation			
IL-1*α*	0 (0–51.7)	6.8 (0–40.7)	∗∗
IL-1RA	39.7 (0–374.9)	34.7 (0–230.9)	ns
TNF-*α*	4.2 (0–15.2)	9.6 (0–60.5)	∗∗∗∗
IFN-*α*	40.6 (0–128.5)	23.6 (3.9–157.9)	ns
IL-6	0 (0–23.4)	6.6 (0–41.4)	∗∗∗∗
Th1 related			
IFN-*γ*	9.3 (0–35.2)	12.7 (0–60.8)	ns
IL-12p40	31.4 (0–140.6)	14 (0–249)	ns
IL-12p70	13.5 (0–73.9)	6.7 (0–58.2)	ns
Bone marrow derived			
IL-7	11 (0–34.9)	9.6 (0–25.4)	ns
GM-CSF	7.6 (0.8–23.0)	5.1 (0–49.2)	ns
G-CSF	144.1 (19.3–458.0)	129.4 (0–310.4)	ns
Stromal, angiogenic			
VEGF	128 (0–344.1)	178.4 (0–1287.3)	ns
EGF	109.8 (42.3–488.2)	109.8 (0–459.5)	ns
Chemokine			
IL-8	2.3 (0–10)	8 (0–61.1)	∗∗∗∗
IP-10	242.6 (97.8–453.3)	1290 (0–9235)	∗∗∗∗
MCP-1	87.1 (48.7–154.5)	128.5 (41.6–280.5)	∗∗∗∗
MIP-1*β*	31.9 (0–51.9)	33.3 (0–58.8)	ns
Eotaxin	38.5 (18.1–112.6)	48 (20.7–133.8)	∗
Matricellular protein			
OPN (ng/mL)	69 (40–118.9)	159 (28.9–256)	∗∗∗∗
Gal-9	195.8 (108–507.6)	377 (0–2181)	∗∗∗∗
Antibody			
Anti-TBGL IgG (U/mL)	2.3 (0.5–37.3)	9.3 (0–64.2)	∗∗
Anti-TBGL IgA (U/mL)	1 (0.2–76)	2.6 (0–57.6)	∗

^*^ *P* < 0.05, ^**^ *P* < 0.01, ^***^ *P* < 0.001, ^****^ *P* < 0.0001, ns: not significant.

TB group versus HC group by Mann-Whitney *U*-test.

**Table 3:** Correlation analysis of IP-10, neutrophil, OPN, and gal-9 with other biomarkers in TB patients.

CBC/clinical parameter/biomarker	IP-10		Neutrophil		OPN		Gal-9	
	*r*	*P*	*r*	*P*	*r*	*P*	*r*	*P*
CBC								
Hemoglobin			−0.375	∗				
Hematocrit			−0.415	∗				
WBC			0.684	∗∗∗∗				
Lymphocyte			−0.931	∗∗∗∗				
Plasma IgG			−0.455	∗				
Clinical parameter								
Loss of appetite	0.466	∗∗						
General activation								
IL-1*α*	0.687	∗∗∗∗						
TNF-*α*							0.39	∗∗
IL-6	0.367	∗					0.309	∗
Th1 related								
IFN-*γ*			0.364	∗				
Bone marrow derived								
GM-CSF							−0.335	∗
Chemokine								
IL-8	0.4635	∗∗			0.44	∗∗	0.4635	∗∗
IP-10					0.422	∗∗	0.295	∗
Matricellular protein								
OPN	0.422	∗∗						
Gal-9	0.393	∗						
Antibody								
Anti-TBGL IgG	−0.423	∗∗						

*P* values of Spearman's rank correlation are expressed as follows: ^*^ *P* < 0.05, ^**^ *P* < 0.01, ^***^ *P* < 0.001, ^****^ *P* < 0.0001.

**Table 4:** Statistic data of ROC analysis of studied biomarkers.

Biomarker	AUC	SE	Cut-off	Sensitivity %	Specificity %	Likelihood ratio
IP-10	0.987	0.009	>342	94.6	93.3	14.1
OPN	0.966	0.027	>94	94.6	93.3	14.1
Neutrophil	0.905	0.036	>57.5	83.8	78.6	3.9
Platelet	0.799	0.055	>339	75.7	80	3.8
TNF-*α*	0.796	0.057	>5.99	81.1	70	2.7
MCP-1	0.786	0.056	>92	75.7	63.3	2.1
WBC	0.784	0.057	>7.5	81.1	70	2.7
Gal-9	0.766	0.063	>258	75.7	80	3.8
IL-8	0.752	0.063	>3.5	75.7	76.7	3.2
Anti-TBGL IgG	0.726	0.063	>4	67.6	70	2.3
Anti-TBGL IgA	0.667	0.069	>1.7	70.3	66.7	2.1
